# Malaysian Tualang Honey Inhibits Hydrogen Peroxide-Induced Endothelial Hyperpermeability

**DOI:** 10.1155/2019/1202676

**Published:** 2019-08-18

**Authors:** Kogilavanee Devasvaran, Jun Jie Tan, Chin Theng Ng, Lai Yen Fong, Yoke Keong Yong

**Affiliations:** ^1^Department of Human Anatomy, Faculty of Medicine and Health Sciences, Universiti Putra Malaysia, 43400 Serdang, Selangor, Malaysia; ^2^Advanced Medical and Dental Institute, Universiti Sains Malaysia, Bertam, 13200 Kepala Batas, Penang, Malaysia; ^3^Physiology Unit, Faculty of Medicine, AIMST University, 08100 Bedong, Kedah, Malaysia; ^4^Department of Pre-clinical Sciences, Faculty of Medicine and Health Sciences, Universiti Tunku Abdul Rahman, 43000 Kajang, Selangor, Malaysia

## Abstract

Malaysian Tualang honey (TH) is a known therapeutic honey extracted from the honeycombs of the Tualang tree (Koompassia excelsa) and has been reported for its antioxidant, anti-inflammatory, antiproliferative, and wound healing properties. However, the possible vascular protective effect of TH against oxidative stress remains unclear. In this study, the effects of TH on hydrogen peroxide- (H_2_O_2_-) elicited vascular hyperpermeability in human umbilical vein endothelial cells (HUVECs) and Balb/c mice were evaluated. Our data showed that TH concentrations ranging from 0.01% to 1.00% showed no cytotoxic effect to HUVECs. Induction with 0.5 mM H_2_O_2_ was found to increase HUVEC permeability, but the effect was significantly reversed attenuated by TH (*p* < 0.05), of which the permeability with the highest inhibition peaked at 0.1%. In Balb/c mice, TH (0.5 g/kg-1.5 g/kg) significantly (*p* < 0.05) reduced H_2_O_2_ (0.3%)-induced albumin-bound Evans blue leak, in a dose-dependent manner. Immunofluorescence staining confirmed that TH reduced actin stress fiber formation while increasing cortical actin formation and colocalization of caveolin-1 and *β*-catenin in HUVECs. Signaling studies showed that HUVECs pretreated with TH significantly (*p* < 0.05) decreased intracellular calcium release, while sustaining the level of cAMP when challenged with H_2_O_2_. These results suggested that TH could inhibit H_2_O_2_-induced vascular hyperpermeability in vitro and in vivo by suppression of adherence junction protein redistribution via calcium and cAMP, which could have a therapeutic potential for diseases related to the increase of both oxidant and vascular permeability.

## 1. Introduction

Vascular diseases are among the leading causes of death worldwide, as they are linked to major illnesses such as atherosclerosis, hypertension, and rheumatoid arthritis [1]. These diseases occur upon an alteration in the homeostatic function in the vascular system. Vascular homeostasis is regulated by the endothelial cell monolayer integrity, which is responsible for the impermeable nature of blood vessels. Changes in endothelial integrity compromise vascular permeability, a physiological response commonly seen in inflammation and angiogenesis [1]. In recent years, growing evidence suggests that oxidative stress can contribute to increased vascular permeability via actin reorganization and Cav-1-associated dissociation of *β*-catenin [2].

Oxidative stress is defined as an imbalance between oxidants and antioxidants in cells due to overproduction of reactive oxygen species (ROS). ROS is also formed during normal cellular metabolism but is highly unstable due to its incomplete reduction of molecular oxygen. At physiological levels, ROS plays a dynamic role in modulating several signaling pathways, related to cell differentiation and growth [3]. Previous studies have shown that hydrogen peroxide (H_2_O_2_), generated by endothelial cells in response to inflammatory stimuli, increase paracellular permeability by promoting the loss of cell-cell adhesion and activation of actin-myosin-based cell retraction [1].

Antioxidants present in traditional medicine have been found to possess potent medicinal properties. Most countries have their own traditional remedies in treating various illnesses with minimal or no known side effects. In Malaysia, the Tualang tree (Koompassia excelsa), the tallest tree in Peninsular Malaysia with an average height of 265 ft, has gained popularity over the years for Tualang honey (TH), the natural product harvested from the honeycombs produced by *Apis dorsata* (giant rock bees) [4]. This therapeutic honey has been reported having the highest phenolic, flavonoid, and ascorbic acid content [5, 6] with an acidic nature at a pH between 3.2 and 4, which makes it bactericidal [7].

Currently, TH is widely studied for its beneficial properties, including promoting wound healing, antibacterial effects, and improved functions of human corneal epithelial cells [8–10]. Furthermore, TH also exhibits cardioprotective effect through ameliorating oxidative stress [11]. Therefore, this study is aimed at investigating the protective effects of Malaysian TH on H_2_O_2_-induced vascular dysfunction as well as its mechanism of action by elucidating the signaling pathway.

## 2. Materials and Methods

### 2.1. Cell Culture

The EndoGRO™ human umbilical vein endothelial cells (HUVECs) (Merck KGaA, Darmstadt, Germany) were cultured in an EndoGRO-LS Complete Culture Media Kit consisting of EndoGRO Basal Medium and its Supplement Kit (Merck KGaA, Darmstadt, Germany). Cells were grown in the incubator, supplemented with 5% CO_2_ at 37°C. Cells were passaged when reaching approximately 80% confluence by using 0.05% trypsin (Biowest) to dissociate the cells. Passage 3-4 HUVECs were used to conduct all the experiments to maintain its originality.

### 2.2. Preparation of Malaysian Tualang Honey Solution

The Tualang honey (TH) used in this study was presented to us by Universiti Sains Malaysia (USM), where the source is from the Federal Agriculture Marketing Authorities of Malaysia (FAMA). TH solutions were prepared right before testing by diluting it to 10% (*v*/*v*) with cultured medium and sterilized by filtering it using a syringe filter (0.2 *μ*m), followed by diluting it further to the required concentrations for cell culture [10]. In the animal study, TH was diluted in normal saline to concentrations 0.5 g/kg, 1.0 g/kg, and 1.5 g/kg based on a previous study [12] prior to testing.

### 2.3. Cell Viability Assay

HUVECs were seeded at 1 × 10^4^ cells/well in a 96-well plate and kept in the incubator supplemented with 5% CO_2_ at 37°C. The following day, culture medium containing the desired concentration of TH (0.001%-10.000%) was added and incubated for 24 h. Then, the medium was replaced by complete medium, to which the Thiazolyl blue tetrazolium bromide or MTT (Amresco, Ohio, USA) was at a final concentration of 0.4 mg/ml. After 4 h of incubation at 37°C in the dark, the medium was removed and the formazon product was dissolved in 100 *μ*l of dimethylsulfoxide (DMSO). The absorbance was measured at 570 nm with a plate reader (SoftMax 5.0, VersaMax ELISA Microplate Reader, USA). Experiments were performed in triplicates and three independent tests. Cell viability was expressed as the percentage of formazon absorbance [13].

### 2.4. In Vitro Vascular Permeability Assay

The permeability of HUVEC monolayer was determined using an in vitro vascular permeability kit (96-well) (Merck KGaA, Darmstadt, Germany) and according to the method described by Yong et al. [14] with a slight modification. Briefly, cells were seeded at 5 × 10^4^ cells/well onto collagen-coated inserts for 72 h. Cells were pretreated with TH at a concentration of 0.01-1.0% for 4 h, and endothelial hyperpermeability was induced with H_2_O_2_ at a concentration of 0.5 mM, followed by addition of FITC-dextran. The plate was incubated for 1 h, and to stop the reaction, the inserts were moved to another well. The fluorescent intensity was measured using a fluorescence microplate reader (Tecan 200, Infinite, Männedorf, Zürich, Switzerland) with an excitation wavelength of 485 nm and emission of 535 nm. Permeability is given as the quantity of FITC-dextran passing from the insert into the receiver well.

### 2.5. Immunofluorescence Staining of Actin Cytoskeleton

The immunofluorescence staining of filamentous actin (F-actin) was conducted according to the methods reported previously [15]. Briefly, HUVECs were cultured on fibronectin-coated round coverslips at 1.5 × 10^5^ cells/well for 4 d at 37°C to reach confluence. After the indicated treatment period, cells were fixed with 3.7% paraformaldehyde (PFA) for 10 min, permeabilised with 0.1% Triton X-100 for 5 min, and stained for F-actin with fluorescein phalloidin (1 : 100, in methanol) for 20 min at room temperature. Next, cells were washed with PBS, and cell nuclei were stained with 4,6-diamidino-2-phenylindole (DAPI; 0.5 *μ*g/ml, in PBS) for 3 min. The coverslips were mounted with a SlowFade Diamond antifade agent, and all the images were captured using a polarizing microscope (BX-51, Olympus, Japan) and processed with Cell^F software (Olympus, Japan).

### 2.6. Immunofluorescence Staining of Cav-1 and *β*-Catenin

HUVECs were grown, treated, and induced as described above. Subsequently, cells were fixed with 3.7% paraformaldehyde for 20 min, permeabilised with 0.10% Triton X-100 for 15 min, and blocked with 2% BSA for 1 h. A primary antibody for *β*-catenin (rabbit anti-*β*-catenin; 5 *μ*g/ml, in 2% BSA) was stained and incubated overnight at 4°C; a secondary antibody (tetramethylrhodamine goat anti-rabbit IgG; 5 *μ*g/ml, in 2% BSA) was then exposed for 2 h at room temperature. Next, a primary antibody for caveolin-1 (mouse anti-Cav-1; 5 *μ*g/ml, in 2% BSA) was stained and incubated overnight at 4°C; a secondary antibody (FITC-goat anti-mouse IgG; 1 : 50, to 2% BSA) was stained for 2 h at room temperature. The nucleus staining with DAPI, mounting of coverslip, and capturing of image were all conducted as described above.

### 2.7. Measurement of Intracellular Calcium

Intracellular calcium was measured using the Fluo-4 Direct™ Calcium Assay Kit (Molecular Probes, Oregon, USA) according to the manufacturer's protocol. Briefly, HUVECs were plated at 5 × 10^4^cells/well and incubated overnight in 5% CO_2_ at 37°C. The following day, the medium was substituted with a Fluo-4 Calcium assay reagent (1 : 1, to culture medium) in the dark and incubated for 30 min. Then, cells were pretreated with TH for 4 h and induced with H_2_O_2_ for 1 h. The fluorescence intensity of the supernatant was measured using a fluorescence microplate reader (Tecan 200, Infinite, Männedorf, Zürich, Switzerland) with an excitation wavelength of 494 nm and emission of 516 nm. The amount of intracellular calcium measured was proportional to the fluorescence intensity.

### 2.8. Measurement of Cyclic Adenosine Monophosphate (cAMP)

The cAMP level was quantified using the Direct cAMP kit (ADI-900-066; Enzo Life Sciences, New York, USA). HUVECs were cultured, treated, and induced, and lysated cells (sample) were prepared as above. Samples were added to appropriate antibody-coated wells. cAMP conjugated to alkaline phosphatase (blue solution) was added prior to the rabbit polyclonal antibody (yellow solution). The antibody was left to bind to the cAMP in the sample. Subsequently, pNpp substrate was added and catalyzed by alkaline phosphatase to produce a yellow colour. Next, stop solution was added to stop the colour development, and the absorbance was measured at 405 nm with a plate reader (SoftMax 5.0, VersaMax ELISA Microplate Reader, USA). The level of cAMP is indirectly proportional to the signal produced (absorbance).

### 2.9. Experimental Animals

A total of 36 male Balb/c mice (*n* = 6/group) in the weight range of 20 to 25 g were bought from the Faculty of Veterinary, Universiti Putra Malaysia (Malaysia), and housed at the Animal House in the Faculty of Medicine and Health Sciences in 12 h dark-light condition (25 ± 2°C) with access to food and water ad libitum. The experimental protocol carried out was approved by University Putra Malaysia, Institutional Animal Care and Use Committee (IACUC), with the AUP No. R011/2015.

### 2.10. Miles Assay

Vascular leak was measured using the Miles assay by quantifying the extravasation of albumin-bound Evans blue into the interstitium from the vasculature of male Balb/c mice [16]. TH at 0.5, 1.0, and 1.5 g/kg was given orally for seven days and 40 min before H_2_O_2_ injection of the seventh day. Another group of mice was orally administered with 35 mg/kg of Trolox as a standard reference. The untreated control and disease groups received only normal saline. On the seventh day, the dorsal fur of the mice was removed using depilatory cream (Veet®, Reckitt Benckiser, UK). Evans blue (Santa Cruz Biotechnology, USA, 0.5% in PBS) was administered via the lateral tail vein and left to circulate for 30 min. Subsequently, H_2_O_2_ was injected intradermally in the dorsal skin. Mice were sacrificed after 10 min, and skin patches from the injection sites were removed and incubated in formamide at 55°C for 24 h. Extracted Evans blue was measured using a spectrophotometer (SoftMax 5.0, VersaMax ELISA Microplate Reader, USA) at 620 nm. The amount of dye extracted was expressed using the formula reported by Radu and Chernoff [17].

### 2.11. Statistical Analysis

Three independent tests were carried out for all experiments (triplicate). All data were expressed as the mean ± standard error of the mean (SEM), and comparisons between groups were analyzed using one-way analysis of variance (ANOVA) with a post hoc analysis using Dunnett's test. A *p* value less than 0.05 (*p* < 0.05) was considered significant.

## 3. Results

### 3.1. TH Is Not Cytotoxic to HUVECs

**Figure 1:**
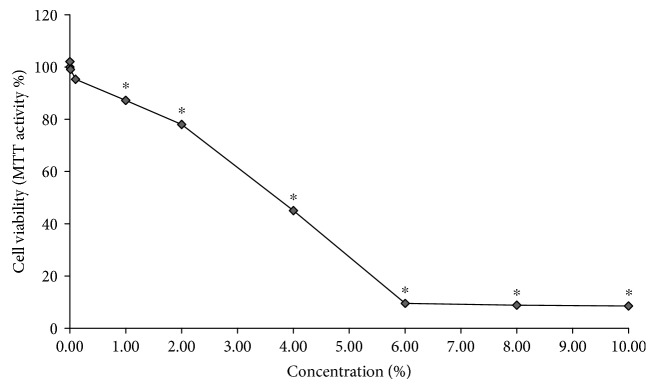
HUVEC viability after treatment with 0.001%-10.00% TH for 24 h. Data were generated from three independent experiments and expressed as the mean ± SEM where ^∗^*p* < 0.05 is considered significant versus the no treatment control.

To study the cytotoxicity of TH, HUVECs were treated with 0.001%, 0.01%, 0.10%, 1.00%, 2.00%, 4.00%, 6.00%, 8.00%, and 10.00%. TH and the percentage of viable cells were assessed after 24 h. As reported in Figure 1, TH was not cytotoxic to HUVECs at concentrations below 1.00% (cell viability of >87%). However, at 2.00% of TH, HUVEC viability was significantly reduced to 78.01% as compared to the untreated control (*p* < 0.05), and the calculated LD_50_ of TH was 3.7%.

### 3.2. TH Protects against Endothelial Barrier Disruption Induced by H_2_O_2_

**Figure 2:**
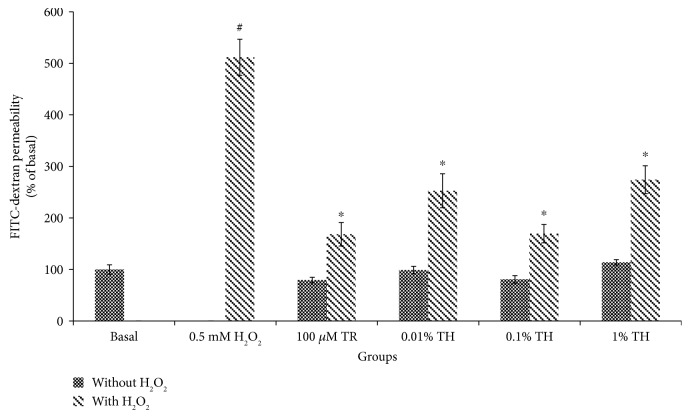
Quantification of FITC-dextran in HUVECs. The permeability (% of basal) of the cells treated with TH with and without the H_2_O_2_ group. Values are expressed as the mean ± SEM from three independent experiments. Hash (#) represents the significant difference compared with the basal group (untreated and with media only), *p* < 0.05; asterisk (∗) represents the significant difference compared to the H_2_O_2_ group, *p* < 0.05. Basal = untreated; TR = Trolox; TH = Tualang honey.

To examine the permeability of HUVECs, the in vitro FITC-dextran-based vascular permeability assay was used. As shown in Figure 2, the permeability in HUVECs treated with TH was not significantly different (*p* > 0.05) from the control, suggesting that TH itself did not cause endothelial hyperpermeability. However, the permeability of HUVECs increased by 5-fold upon H_2_O_2_ induction to 511.85 ± 35.04% from the control 100.00 ± 9.16%. Pretreatment with TH at all tested concentrations significantly attenuated the increased permeability elicited by H_2_O_2_ (*p* < 0.05). The maximal inhibition of permeability was achieved with 0.10% TH, achieving a reduction to 169.69 ± 17.79% (*p* < 0.05).

### 3.3. TH Inhibits H_2_O_2_-Stimulated Actin Remodeling in HUVECs

**Figure 3:**
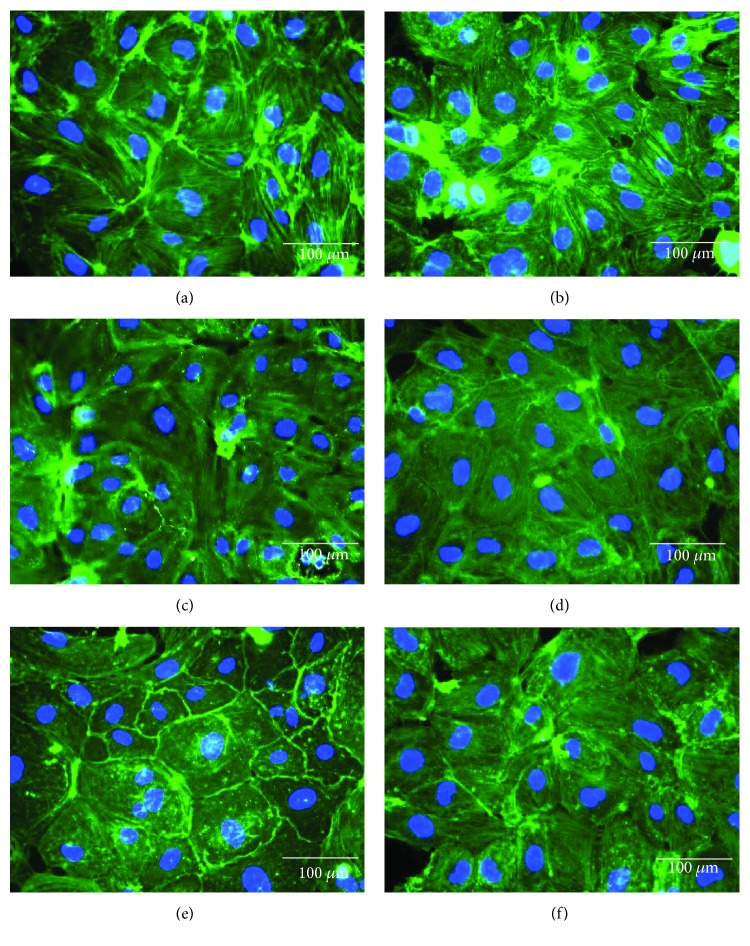
Effect of HUVECs pretreated with TH on H_2_O_2_-induced cytoskeletal remodeling and gap formation. Actin cytoskeleton (green) is shown merged with its nucleus (blue) stained with DAPI. Representative images were taken from one of three independent experiments at 400x magnification. Merged images were generated with Olympus BX-51 Cell-F imaging software. Images represent basal (a); H_2_O_2_ at 0.5 mM (b); and pretreatments+inducer: TH 0.01%+H_2_O_2_ (c), TH 0.10%+H_2_O_2_ (d), TH 1.00%+H_2_O_2_ (e), and TR+H_2_O_2_ (f). Basal = untreated; TH = Tualang honey; TR = Trolox.

As illustrated in Figure 3, a prominent cortical actin bundle was formed in control HUVECs, with a low level of stress fibers. In the H_2_O_2_ group (Figure 3(b)), stress fiber formation was dominant and led to gaps between cells. In groups of TH, at 0.01% (Figure 3(c)), the presence of stress fibers was more compared to the cortical actin; at 0.10% (Figure 3(d)), a suppression in the stress fiber formation was observed, which led to a minimal gap formation among cells, similar to the positive control, Trolox (Figure 3(f)); and at 1.00% (Figure 3(e)), the cells showed generally fewer and thinner stress fibers, also with some gaps formed between cells. Although H_2_O_2_ exposure caused the induction of cytoplasmic stress fibers and a less prominent cortical actin bundle, TH managed to block the effects of H_2_O_2_.

### 3.4. TH Inhibits H_2_O_2_-Induced Cav-1-Mediated Dissociation of *β*-Catenin in HUVECs

**Figure 4:**
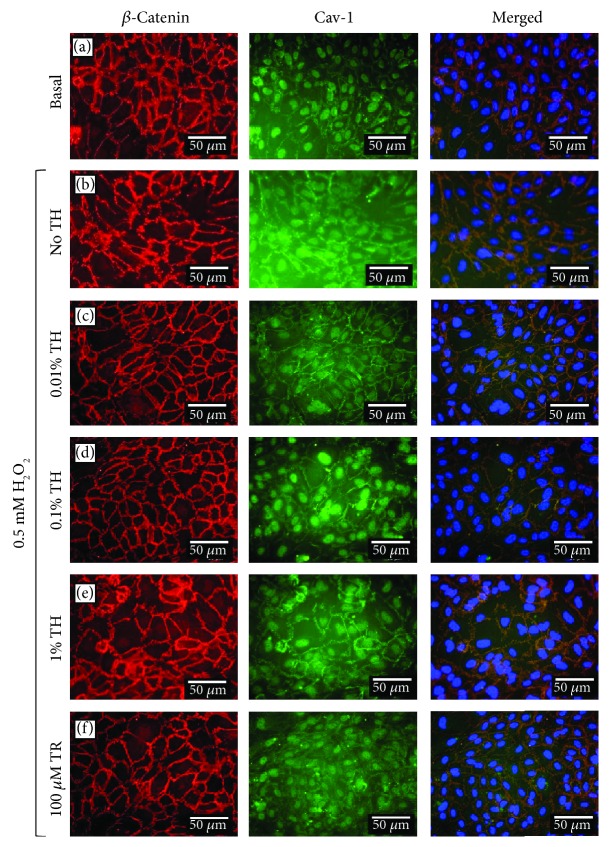
Localization of *β*-catenin and caveolin-1 in HUVECs pretreated with TH and induced with H_2_O_2_. Cellular localization of *β*-catenin (red) and Cav-1 (green) is shown merged with nucleus (blue) stained with DAPI. Merged images were generated with Olympus BX-51 Cell-F imaging software, whereby a third pseudocolour channel (orange) was used to show colocalization of red and green pixels. Representative images were taken from one of three independent experiments. Basal = untreated; TH = Tualang honey; TR = Trolox.

To understand the effect of TH on H_2_O_2_-induced endothelial barrier disruption and vascular permeability, the changes of Cav-1 and *β*-catenin colocalization in HUVECs were observed.  Figure 4 shows that H_2_O_2_ decreased the colocalization of Cav-1 and *β*-catenin at the cell borders which coupled with the dissociation of barrier integrity that showed a rope ladder-like pattern as compared to the basal group. However, HUVECs pretreated with TH showed a marked increase in the association between Cav-1 and *β*-catenin. Interestingly, the greatest extent of colocalization between Cav-1 and *β*-catenin was observed at 0.10% TH, of which the extent of inhibition of H_2_O_2_-induced barrier disruption was similar to that of the positive control (Trolox).

### 3.5. TH Inhibits H_2_O_2_-Induced Intracellular Calcium Formation

**Figure 5:**
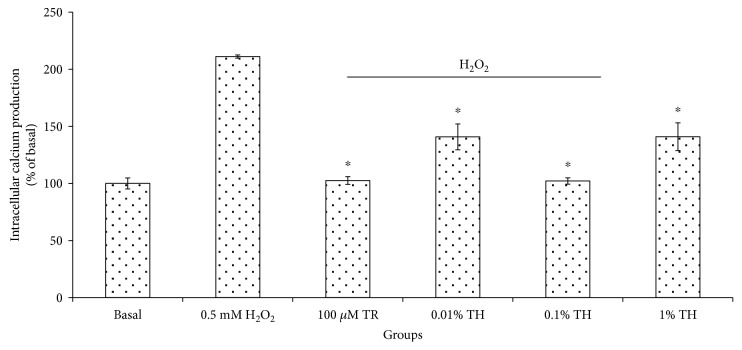
Percentage of intracellular calcium production in HUVECs pretreated with TH and induced with H_2_O_2_. The intracellular calcium (% of basal) of cells treated with TH shows a significant difference (^∗^*p* < 0.05) with the H_2_O_2_ group. Values are expressed as the mean ± SEM from three independent experiments. Asterisk (∗) represents the significantly different groups with the H_2_O_2_ group. Basal = untreated; TR = Trolox; TH = Tualang honey.

To detect the intracellular calcium, the Fluo-4 calcium probe was used measured using a fluorescent plate reader. As shown in Figure 5, H_2_O_2_ stimulation resulted in a significant (*p* < 0.05) increase in the intracellular calcium by approximately twofold 211.11 ± 1.39% as compared to the basal 100.00 ± 4.81%. Pretreatment of TH significantly (*p* < 0.05) reduced the excessive intracellular calcium release with the highest percentage of intracellular calcium suppression of 102.12 ± 2.76% exhibited by TH at 0.10% and was comparable to the basal group.

### 3.6. TH Maintains cAMP Levels

**Figure 6:**
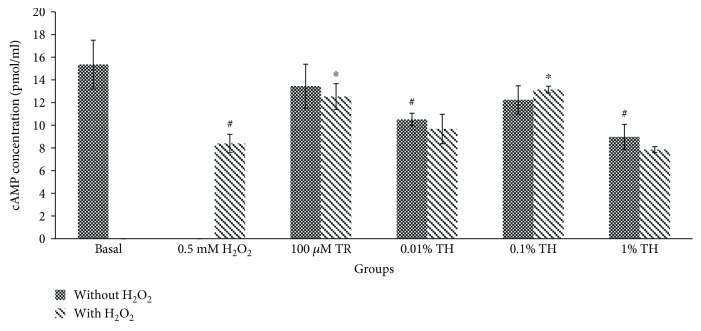
Amount of cAMP production in HUVECs. The cAMP concentration (pmol/ml) of cells treated with TH alone or induced with H_2_O_2_. Values are expressed as the mean ± SEM from three independent experiments. Hash (#) represents the significant difference compared to the untreated group (basal), *p* < 0.05; asterisk (∗) represents the significant difference compared to the H_2_O_2_ alone group, *p* < 0.05. Basal = untreated; TR = Trolox; TH = Tualang honey.

To elucidate the mechanism of TH barrier protective effect, cAMP in HUVECs was quantified with and without induction by H_2_O_2_. As shown in Figure 6, exposure to H_2_O_2_ significantly (*p* < 0.05) decreased the production of cAMP in HUVECs by half as compared to the control group (from 15.36 ± 2.15 pmol/ml to 8.39 ± 0.81 pmol/ml). Although groups with TH alone at 0.01 and 1.00% significantly reduced the cAMP level in HUVECs (*p* < 0.05), the cAMP level in HUVECs treated with 0.10% TH showed no significant difference compared to control group. Interestingly, only HUVECs pretreated with 0.10% TH significantly (*p* < 0.05) attenuated the reduction in cAMP production to 12.23 ± 1.24 pmol/ml as compared to the untreated control, when both were challenged by H_2_O_2_. Similarly, in Trolox-treated HUVECs, a higher cAMP level was found when the cells were challenged with H_2_O_2_.

### 3.7. TH Protects against H_2_O_2_-Induced Vascular Leakage in Balb/c Mice

**Figure 7:**
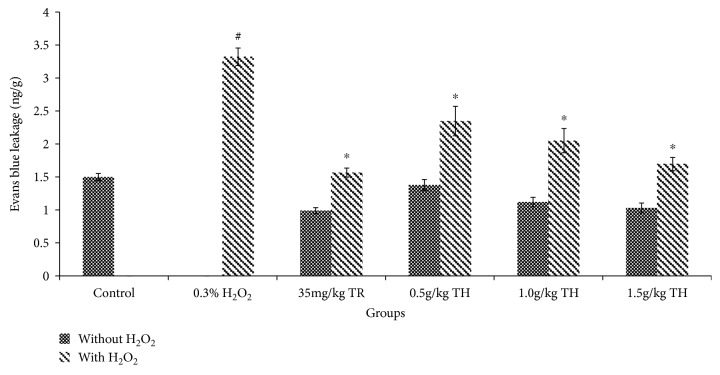
Quantitation of Evans blue extravasation in the skin of Balb/c mice. Mice treated with TH showed no significant difference (*p* > 0.05) compared with the untreated control group. ^#^*p* < 0.05 vs. the control group; ^∗^*p* < 0.05 vs. the H_2_O_2_ only group. Data were expressed as the mean ± SEM (*n* = 6). Control = untreated; TR = Trolox; TH = Tualang honey.

To test the protective effect of TH on endothelial barrier function in vivo, TH was pretreated in mouse models, and the vascular leakage, measured by Evans blue in tissues, was determined. All concentrations of TH including the reference drug (Trolox) showed no significant difference compared to the control group (basal level) (Figure 7). H_2_O_2_ caused a significant increase in dye leakage by almost 100% compared to the control group (from 1.50 ± 0.05 ng/g to 3.32 ± 0.13 ng/g) (*p* < 0.05). However, there was a significant decrease in dye leakage in mice pretreated with TH at 0.5, 1.0, and 1.5 g/kg when compared to H_2_O_2_ alone with a suppression rate of 29%, 38%, and 49%, respectively (*p* < 0.05).

## 4. Discussion

Tualang honey (TH) has gained attention for its various properties such as wound healing [8], antibacterial properties [18], and antiproliferative properties [6]. However, the effects of TH at a cellular level and its potential as a vascular protective agent have not been studied. In this study, we evaluated the protective effect of TH on oxidative stress-induced increased endothelial permeability.

Exposure to a high level of oxidative stress such as hydrogen peroxide (H_2_O_2_) can cause contraction and separation of endothelial cells and results in increased endothelial permeability and exudation of fluid rich in plasma protein at the site of inflammation.

Our data showed that TH was capable to reduce HUVEC hyperpermeability when the cells were challenged with H_2_O_2_, with a concentration of only 0.10% of TH to yield the most potent inhibition rate similar to the basal permeability level. Similarly, *in vivo*, mice pretreated with TH also significantly (*p* < 0.05) reduced dye leakage from the microvessels following H_2_O_2_. Hence, both data (*in vitro* and *in vivo*) strongly suggest that TH can effectively suppress the exudative phase of acute inflammation, probably due to its high content of phenolic/flavonoid compounds, and radical scavenging activity [6].

Aghajanian et al. [19] demonstrated that endothelial cells exposed to H_2_O_2_ led to the remodeling of the actin filament, disrupted the cortical bond necessary for barrier integrity, increased intracellular tension and paracellular gap formation, and therefore, increased permeability. Once again, this process was reversed by TH, with its effect peaked at 0.10%, suggesting that the inhibition of HUVEC hyperpermeability by TH was via maintaining the actin filament, increasing the cortical actin bond which is important in maintaining the barrier integrity, and reducing the intracellular tension, thus leading to minimal intracellular gap formation [20].

Caveolae are abundant in endothelial cells, and they do play a part in vascular permeability. The formation of plasma membrane caveolae is driven by Cav-1 and is brought to the actin cytoskeleton, which regulates the interaction of cells with the extracellular matrix that eventually pulls together and modulates signaling molecules [21]. Cav-1 stabilizes the adherence junctions [22], and colocalization of Cav-1 and *β*-catenin, an adherent junction-associated protein, is important in maintaining the barrier integrity, specifically the interendothelial junctions [2]. Our study showed that H_2_O_2_ disrupts the colocalization between Cav-1 and *β*-catenin at the cell borders and dissociates barrier integrity in a rope ladder-like pattern. These events, again, were prevented by pretreatment of TH. The observation suggested that TH was able to reduce vascular hyperpermeability induced by H_2_O_2_ via increasing the colocalization between Cav-1 and *β*-catenin at the cell borders.

Several studies have shown that when endothelial cells are exposed to agonists like H_2_O_2_ [23], it raises the intracellular calcium concentration that causes increased endothelial permeability [23–25]. In the present study, the increase in the intracellular calcium level in HUVECs induced by H_2_O_2_ (*p* < 0.05) was abolished by TH and the level of intracellular calcium was almost reversed back to its basal level. Such maximal inhibition required only 0.10% of TH, suggesting that TH can reverse endothelial hyperpermeability through inhibiting H_2_O_2_-induced upregulation of intracellular calcium. Future investigation would be aimed at revealing if such suppression caused by TH occurs via inhibiting extracellular calcium influx or intracellular calcium production.

cAMP, a barrier-stabilizing molecule, could antagonize vascular leakage and protect endothelial barrier functions. By elevating cAMP levels, oxidant-induced permeability and edema formation can be reduced [26]. H_2_O_2_ is known to decrease cellular cAMP levels [27]. Another study demonstrated that the formation of cortical actin (F-actin cross linking protein, with cell protective effects) is a cAMP-dependent process [1]. In our study, pretreatment of TH alone on HUVECs reduced the cAMP production (0.01% and 1.0%); however, 0.10% of TH showed no significant difference as compared with the basal group even if there was a slight reduction (Figure 6). Interestingly, HUVECs induced with H_2_O_2_ were protected by TH especially at 0.10%, via maintaining the cAMP level. Low and high concentrations of TH (0.01% and 1.00%, respectively) failed to upregulate the cAMP level. This suggests that TH at its optimal concentration (0.10%) exhibited the maximal effect where it was able to maintain cAMP production which is comparable to the basal group.

Further investigation is needed to elucidate a clear TH-mediated signaling mechanism underlying our observation, e.g., redox-sensitive protein kinases such as mitogen-activated protein kinase (MAPK), using a more sophisticated tool to study the gene and protein expressions, and evaluate the differences in treatment response in animals of different genders to provide better translation insight to warrant clinical study in the future.

## 5. Conclusions

In summary, the present study provided the evidence that TH can inhibit H_2_O_2_-induced vascular permeability *in vivo* and *in vitro*. Such inhibition is via actin cytoskeleton reorganization, localization of *β*-catenin from Cav-1, and reduction of intracellular calcium influx while sustaining the cAMP levels. These discoveries may make a significant contribution to the pathogenesis of oxidant-dependent vascular diseases.

## Data Availability

The data used to support the findings of this study are available from the corresponding author upon request.

## Supplementary Materials

Supplementary Materials Supplementary Materials Qualification of Evans blue extravasation in the skin of Balb/c mice. (a) Control group where mice were only treated with vehicle; (b) group pretreated with 35 mg/kg of TR; (c) group of mice pretreated with 0.5 g/kg of TH; (d) group of mice pretreated with 1.0 g/kg of TH; (e) group of mice pretreated with 1.5 g/kg of TH. Control = untreated; TR = Trolox; TH = Tualang honey.
