# Supplementary material for: Malaysian Tualang Honey Inhibits Hydrogen Peroxide-Induced Endothelial Hyperpermeability
Source: Oxid Med Cell Longev. 2019 Aug 18;2019:1202676. doi: 10.1155/2019/1202676 (PMC6721116; doi:10.1155/2019/1202676)
Supplement: Supplementary Materials — Qualification of Evans blue extravasation in the skin of Balb/c mice. (a) Control group where mice were only treated with vehicle; (b) group pretreated with 35 mg/kg of TR; (c) group of mice pretreated with 0.5 g/kg of TH; (d) group of mice pretreated with 1.0 g/kg of TH; (e) group of mice pretreated with 1.5 g/kg of TH. Control = untreated; TR = Trolox; TH = Tualang honey. [file 1202676.f1.pptx]

## Slide 1
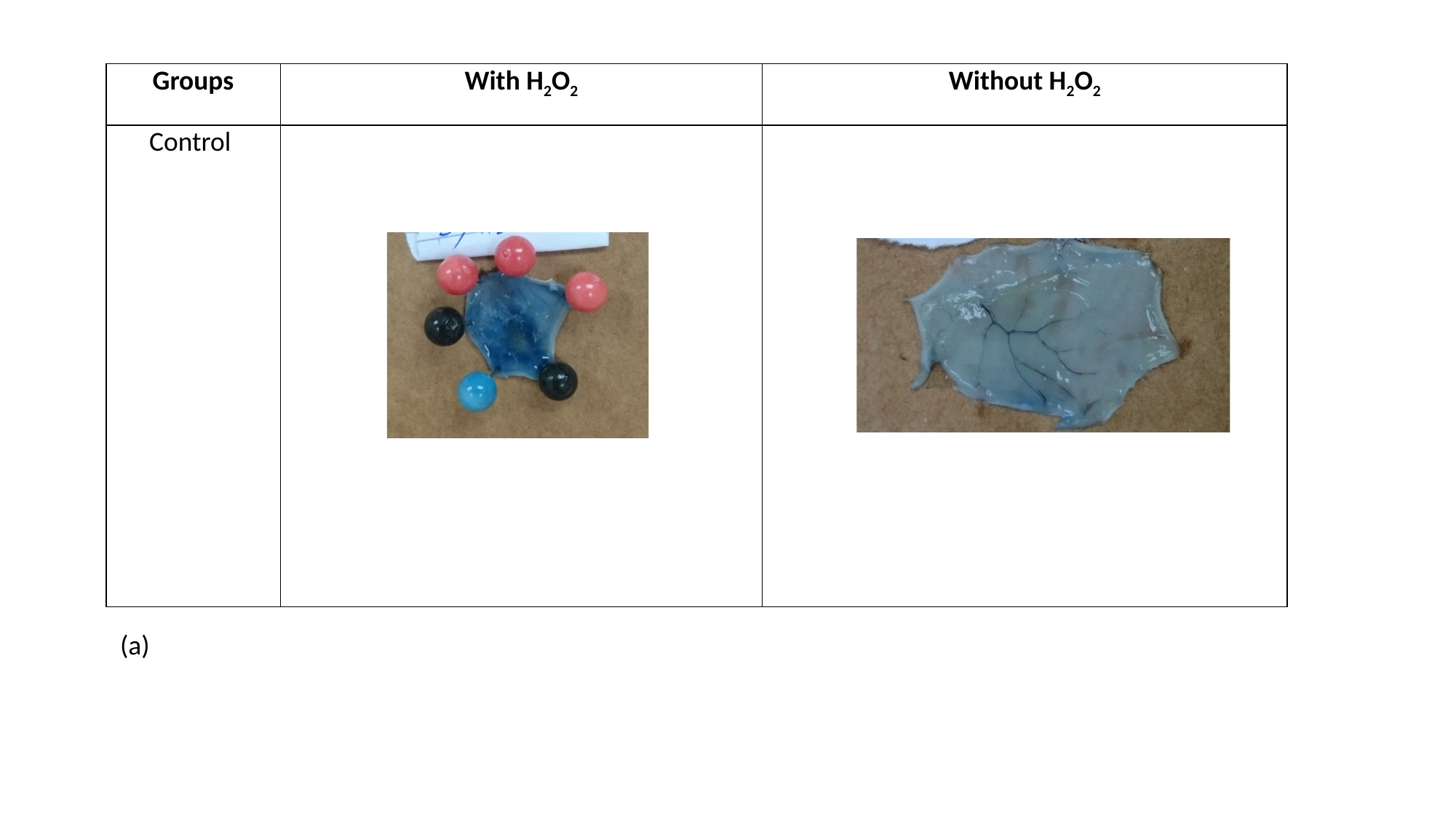

| Groups | With H2O2 | Without H2O2 |
| --- | --- | --- |
| Control | | |
(a)

## Slide 2
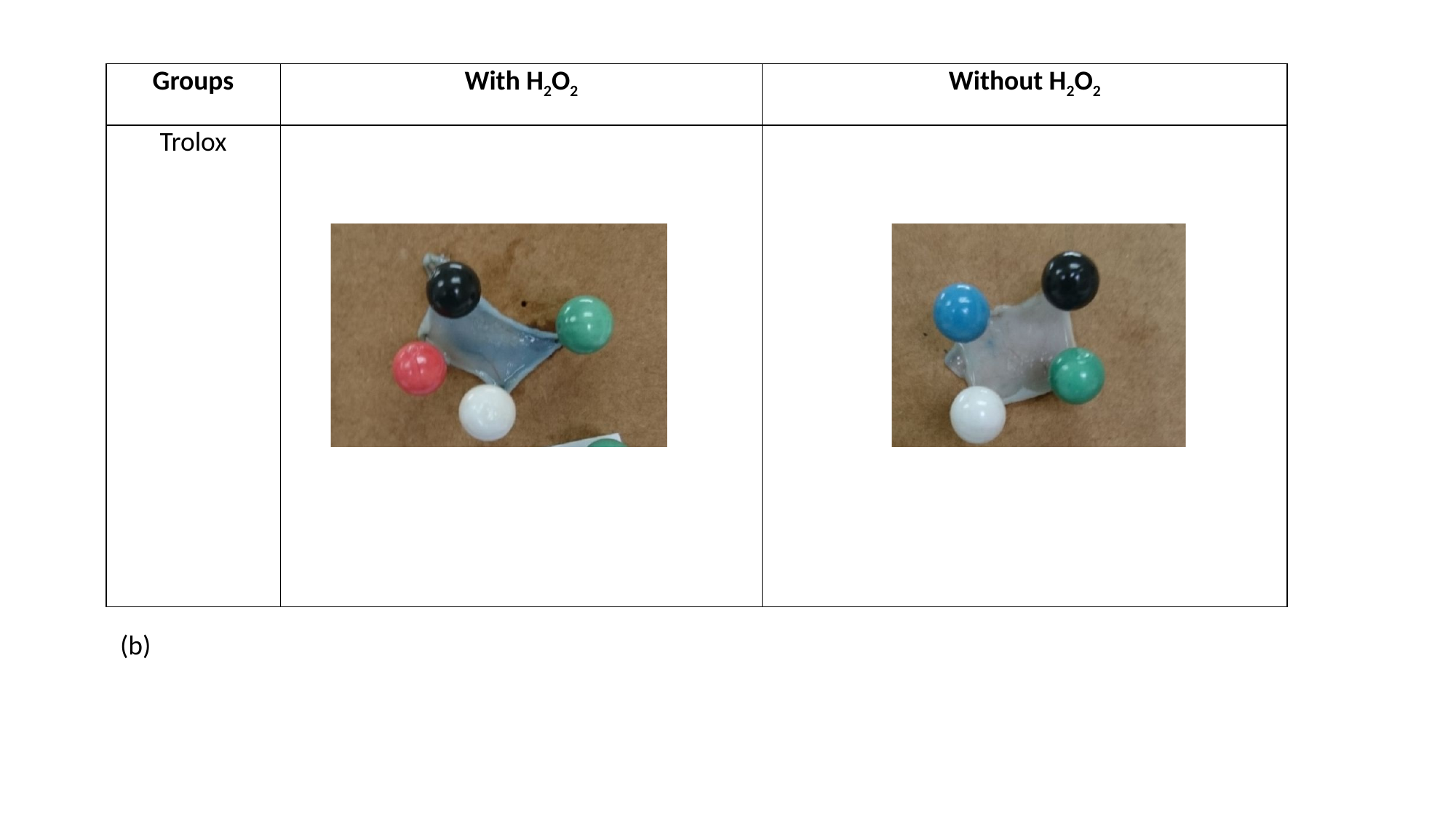

| Groups | With H2O2 | Without H2O2 |
| --- | --- | --- |
| Trolox | | |
(b)

## Slide 3
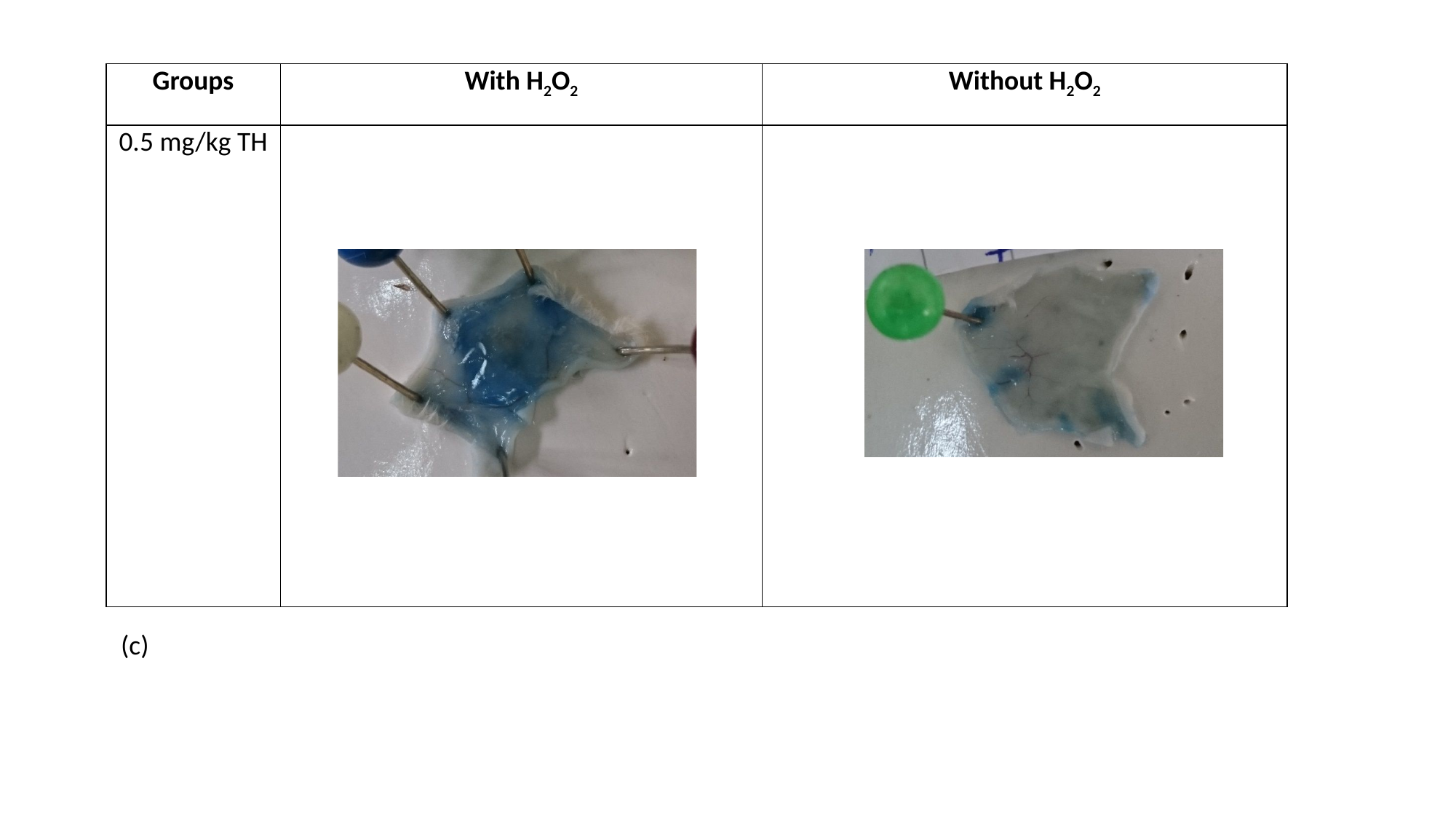

| Groups | With H2O2 | Without H2O2 |
| --- | --- | --- |
| 0.5 mg/kg TH | | |
(c)

## Slide 4
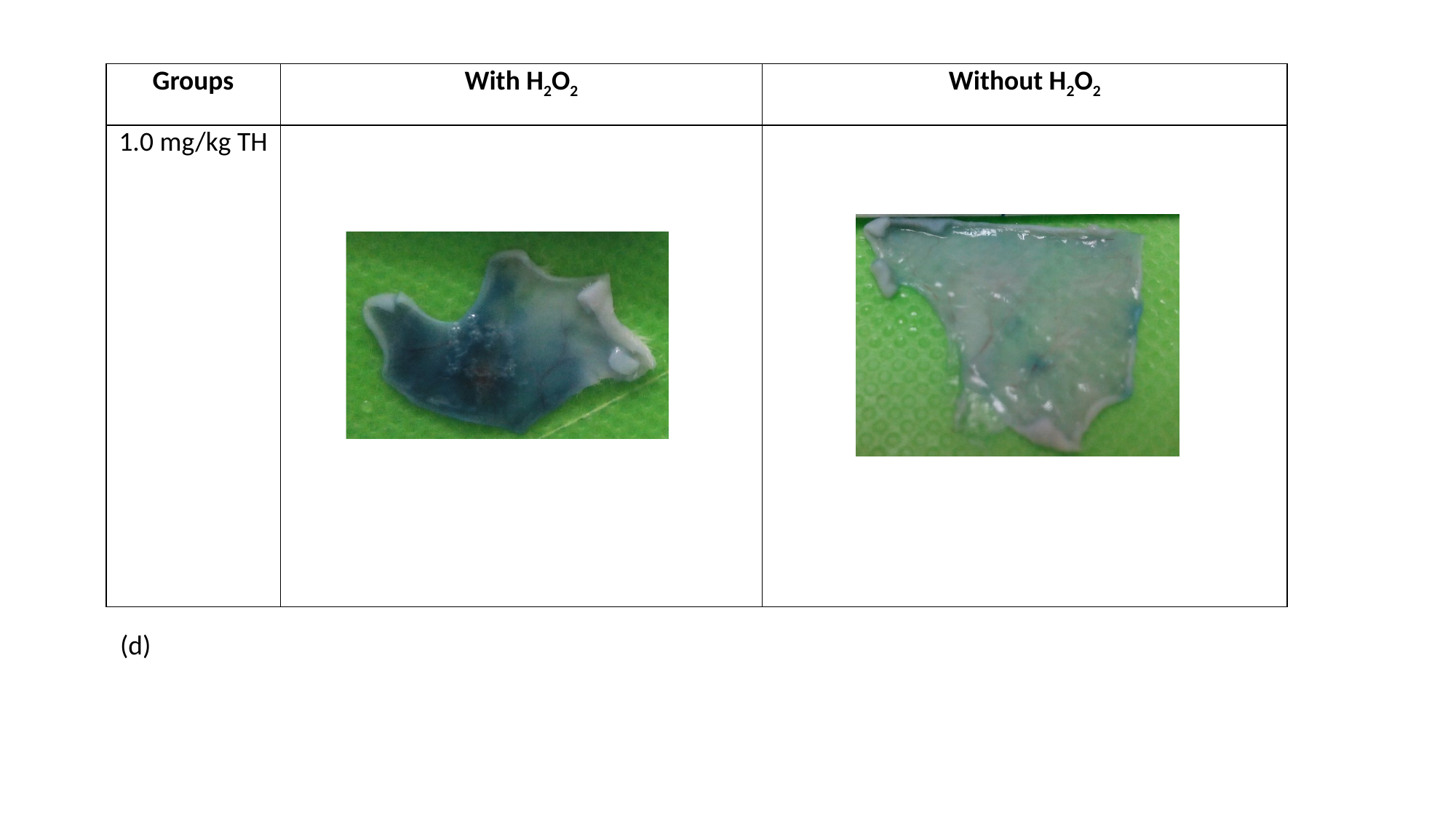

| Groups | With H2O2 | Without H2O2 |
| --- | --- | --- |
| 1.0 mg/kg TH | | |
(d)

## Slide 5
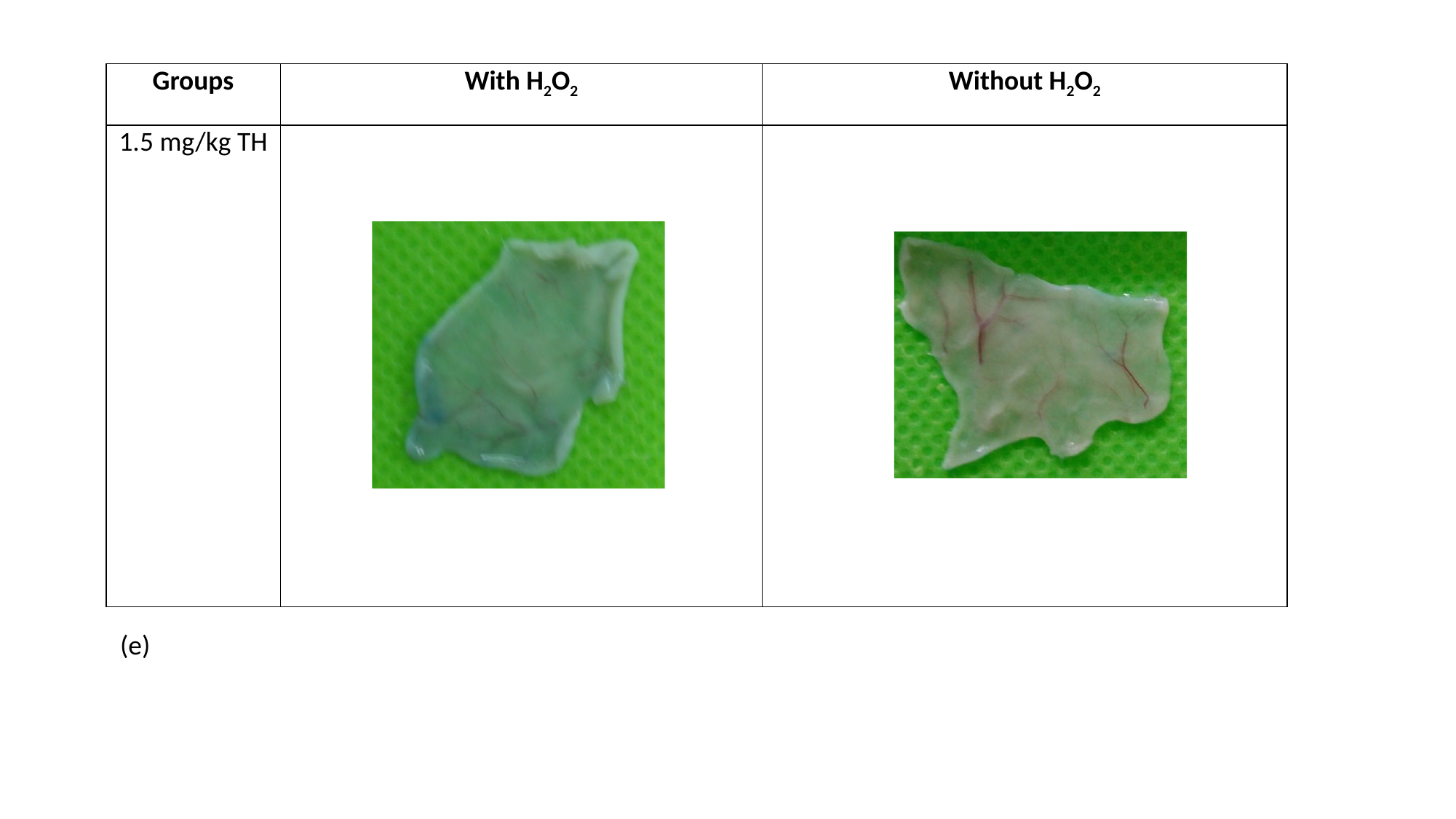

| Groups | With H2O2 | Without H2O2 |
| --- | --- | --- |
| 1.5 mg/kg TH | | |
(e)

## Slide 6
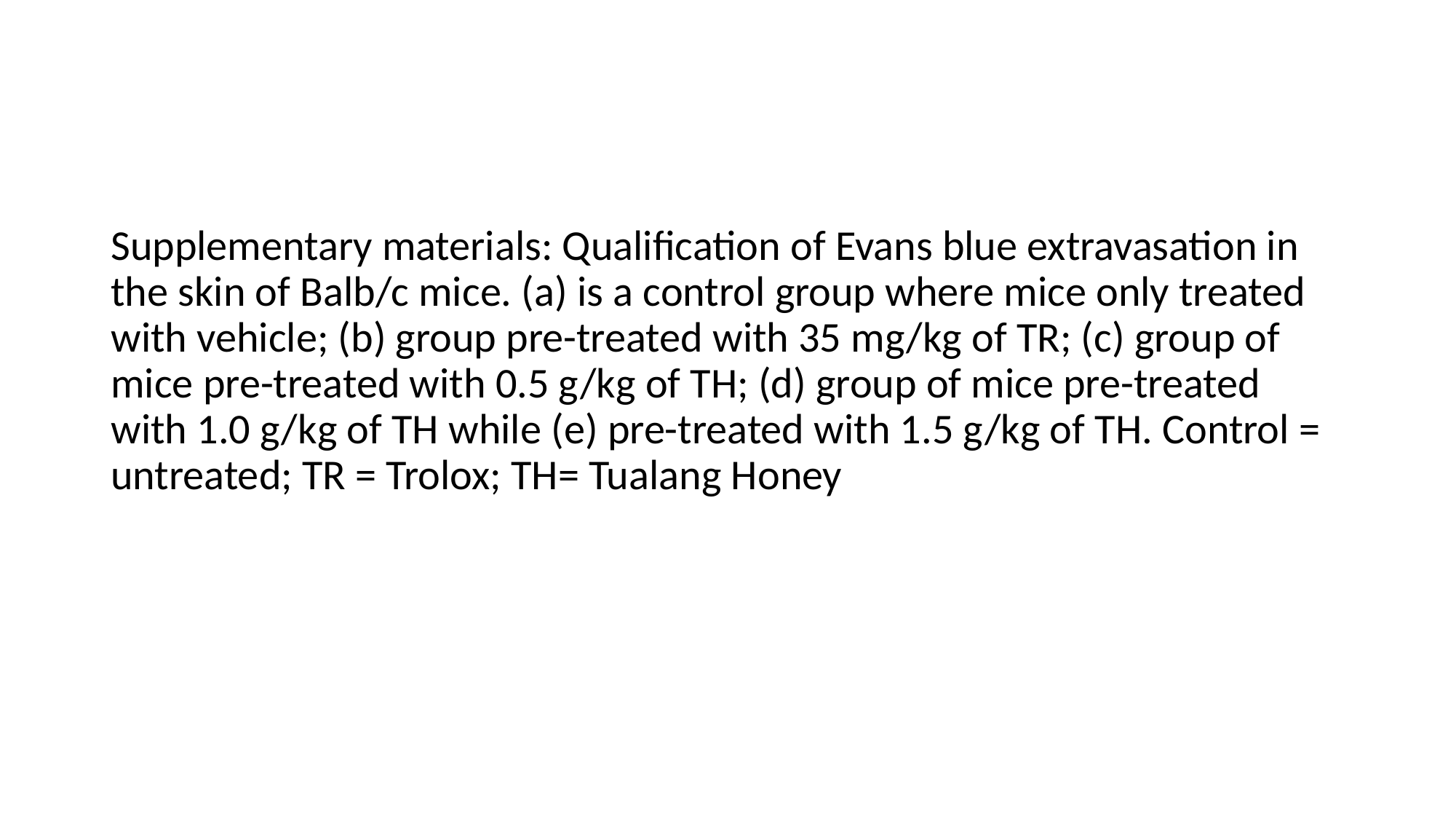

Supplementary materials: Qualification of Evans blue extravasation in the skin of Balb/c mice. (a) is a control group where mice only treated with vehicle; (b) group pre-treated with 35 mg/kg of TR; (c) group of mice pre-treated with 0.5 g/kg of TH; (d) group of mice pre-treated with 1.0 g/kg of TH while (e) pre-treated with 1.5 g/kg of TH. Control = untreated; TR = Trolox; TH= Tualang Honey
